# The effect of LITHIUM in mood improvement in patient with borderline personality disorder; A systematic review

**DOI:** 10.1192/j.eurpsy.2025.1919

**Published:** 2025-08-26

**Authors:** S. Ghorani, M. Zamir

**Affiliations:** 1 Qazvin medical university, 22 bahman hospital, QAZVIN, Iran, Islamic Republic Of

## Abstract

**Introduction:**

Considering the need to conduct studies on the treatment of BPD and considering the high prevalence of this disorder and its negative effect on the quality of life, especially in young age range and possibility of this disorder being in the Bipolar spectrum, it is necessary to investigate the effect of lithium on mood improvement in patients with BPD. So, this study aimed to investigate the effect of lithium on mood improvement in these patients.

**Objectives:**

Due to the lack of an FDA approved treatment, as well as discussed in the introduction, we dicided to compare the effect of lithiumin mood improvement in this disorder with the effect of other mood stabilizers and antipsychotics.

For this reason, we reviewed the published articles on the effects of other mood stabilizers and antipsychotics on improving the mood of this patints and compare them with those related to lithium in order to make better treatment decision.

**Methods:**

This study presents a comprehensive review of the studies conducted related to the effect of lithium in improving mood in BPD patients. We conducted a systematic review based on PRISMA guidelines of published and indexed articles from the following databases;

EMBASE, MEDLINE, Google scholar, SCOPUS.Cochrane Library, PsycINFO.

KEY WORD: Lithium, Mood stabilizer, borderline personality disorder. Of the 131 retrieved articles, 9 included our inclusion criteria.

**Results:**

The review of 9 selected studies showed that lithium is useful in reducing emotional and impulsive behaviors, mood stabilization and suicidal tendencies and was more effective than placebo in preventing recurrence of mood disorders. In the study significant heterogeneity was found between all group of patients which could be due to the difference in the selection of participants and different exposure in the pre-study phase. Quantitative data on participants general health and social functioning were not reported and the direction of effect was the same in all studies.

**Conclusions:**

No studies reported a negative effect for lithium and compared to other mood stabilization, it is more effective in controlling emotional and mood changes and aggression, also its side effects are less if controlled regularly and consistently. However, due to the small number of studies in this field and small sample size in studies, we suggest that more studies be conducted in all age groups.

**Disclosure of Interest:**

None Declared

